# Public Figure Vaccination Rhetoric and Vaccine Hesitancy: Retrospective Twitter Analysis

**DOI:** 10.2196/40575

**Published:** 2023-03-10

**Authors:** Vlad Honcharov, Jiawei Li, Maribel Sierra, Natalie A Rivadeneira, Kristan Olazo, Thu T Nguyen, Tim K Mackey, Urmimala Sarkar

**Affiliations:** 1 Division of General Internal Medicine at Zuckerberg San Francisco General Hospital and Trauma Center University of California San Francisco San Francisco, CA United States; 2 Center for Vulnerable Populations University of California San Francisco San Francisco, CA United States; 3 S-3 Research LLC San Diego, CA United States; 4 Global Health Policy and Data Institute San Diego, CA United States; 5 Department of Family and Community Medicine University of California San Francisco San Francisco, CA United States; 6 Department of Epidemiology & Biostatistics University of Maryland School of Public Health College Park, MD United States; 7 Global Health Program Department of Anthropology University of California San Diego La Jolla, CA United States

**Keywords:** Twitter, anti-vaccination, Biterm Topic modeling, inductive content analysis, COVID-19, social media, health information, vaccination, vaccine hesitancy, infodemiology, misinformation

## Abstract

**Background:**

Social media has emerged as a critical mass communication tool, with both health information and misinformation now spread widely on the web. Prior to the COVID-19 pandemic, some public figures promulgated anti-vaccine attitudes, which spread widely on social media platforms. Although anti-vaccine sentiment has pervaded social media throughout the COVID-19 pandemic, it is unclear to what extent interest in public figures is generating anti-vaccine discourse.

**Objective:**

We examined Twitter messages that included anti-vaccination hashtags and mentions of public figures to assess the connection between interest in these individuals and the possible spread of anti-vaccine messages.

**Methods:**

We used a data set of COVID-19–related Twitter posts collected from the public streaming application programming interface from March to October 2020 and filtered it for anti-vaccination hashtags “antivaxxing,” “antivaxx,” “antivaxxers,” “antivax,” “anti-vaxxer,” “discredit,” “undermine,” “confidence,” and “immune.” Next, we applied the Biterm Topic model (BTM) to output topic clusters associated with the entire corpus. Topic clusters were manually screened by examining the top 10 posts most highly correlated in each of the 20 clusters, from which we identified 5 clusters most relevant to public figures and vaccination attitudes. We extracted all messages from these clusters and conducted inductive content analysis to characterize the discourse.

**Results:**

Our keyword search yielded 118,971 Twitter posts after duplicates were removed, and subsequently, we applied BTM to parse these data into 20 clusters. After removing retweets, we manually screened the top 10 tweets associated with each cluster (200 messages) to identify clusters associated with public figures. Extraction of these clusters yielded 768 posts for inductive analysis. Most messages were either pro-vaccination (n=329, 43%) or neutral about vaccination (n=425, 55%), with only 2% (14/768) including anti-vaccination messages. Three main themes emerged: (1) anti-vaccination accusation, in which the message accused the public figure of holding anti-vaccination beliefs; (2) using “anti-vax” as an epithet; and (3) stating or implying the negative public health impact of anti-vaccination discourse.

**Conclusions:**

Most discussions surrounding public figures in common hashtags labelled as “anti-vax” did not reflect anti-vaccination beliefs. We observed that public figures with known anti-vaccination beliefs face scorn and ridicule on Twitter. Accusing public figures of anti-vaccination attitudes is a means of insulting and discrediting the public figure rather than discrediting vaccines. The majority of posts in our sample condemned public figures expressing anti-vax beliefs by undermining their influence, insulting them, or expressing concerns over public health ramifications. This points to a complex information ecosystem, where anti-vax sentiment may not reside in common anti-vax–related keywords or hashtags, necessitating further assessment of the influence that public figures have on this discourse.

## Introduction

COVID-19 is a member of a large family of viruses called coronaviruses [1]. The virus is spread from person to person through droplets released when an infected person coughs, sneezes, or talks and is less commonly spread by touching a surface with the virus on it and then touching one’s eyes, mouth, or nose [1,2]. COVID-19 was first detected in late December 2019 and was declared a pandemic by the World Health Organization in March 2020 [2,3]. Symptoms typically include fever, malaise, and cough, and some who are infected develop acute respiratory distress syndrome, respiratory failure, organ failure, and even death [2,3].

Even in the context of an ongoing COVID-19 pandemic, anti-vaccination rhetoric persists despite scientific evidence validating the safety and efficacy of vaccines as a critical public health tool [4]. Existing literature indicates that false claims regarding COVID-19 vaccines undermine public trust in ongoing vaccination campaigns, which can lead to greater morbidity and mortality from vaccine-preventable diseases [5]. Social media plays a role in shaping vaccination beliefs, as 7 of every 10 Americans report using a social media platform [6]. For example, Twitter, a popular microblogging platform that allows users to share posts of 280 characters or less, commonly referred to as “tweets,” boasted 290.5 million users in 2019. Twitter has also been identified as a source of misinformation and disinformation about COVID-19 and vaccines [7,8]. An investigation into misinformation warnings on Twitter found that rather than dispelling misinformation, moderation often led to the development of reverberations of one’s own beliefs regardless of the presence of the disclaimer [9]. This is supported by research indicating that social media users heavily relied on social media platforms for COVID-19 information and were unlikely to fact check the information they obtained with a professional [10].

Researchers have previously examined the role public figures on social media have in shaping the public’s health beliefs. Prior studies have found that only a handful of individual accounts can be responsible for disseminating information and misinformation that is then shared or retweeted thousands of times, reaching potentially millions of social media users [11]. Furthermore, a 2013 meta-analysis found that individuals are conditioned to react positively to the advice of celebrities, and that celebrity medical advice can be a contagion that diffuses throughout social networks [12]. Consequently, celebrity anti-vaccination rhetoric can have extensive, deleterious consequences on public health. Notably, public figures may even propagate health misinformation inadvertently. A retrospective Twitter analysis examining the diffusion of misinformation following Hank Aaron’s death found an increase in erroneous claims connecting his death to vaccine misinformation [13].

Many Twitter posts about COVID-19 vaccination reference public figures, but it remains unclear how the discourse surrounding vaccination integrates attitudes and opinions about public figures. It is also undetermined whether the conversation about public figures’ vaccination attitudes is intended to fuel anti-vaccination sentiments. Therefore, we aimed to study Twitter posts about COVID-19 vaccination that specifically mentioned publicly known individuals or groups, while concurrently investigating the themes and sentiments depicted in these associated posts.

## Methods

### Ethics Approval

As this study used deidentified, publicly available social media data, the Institutional Review Board of University of California, San Francisco classified our proposal as exempt from review (IRB 13-12815).

### Procedure

**Figure 1:**
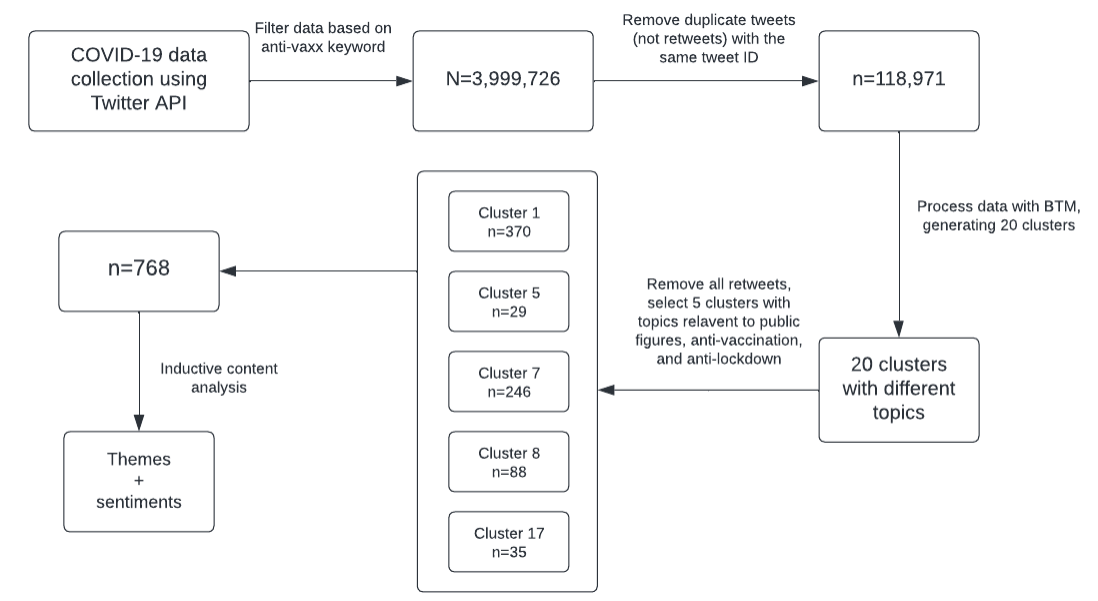
Study design. API: application programming interface; BTM: Biterm Topic Modeling.

We collected publicly available data using Twitter’s application programming interface (API) as seen in previous social media research (Figure 1) [14]. Specifically, the purpose of this study was to identify tweets associated with anti-vax discussion that also included mentions of public figures. Hence, though data on individual Twitter user accounts or handles were collected, they were subsequently removed from the data set prior to the topic modeling phase of the study and were not analyzed or reported other than in the aggregate.

Next, we removed all duplicate Twitter posts and conducted topic exploration using an unsupervised machine learning approach called Biterm Topic modeling (BTM), which thematically groups related Twitter posts into topic clusters [14,15]. We defined Twitter messages as texts—with 280 characters or less—posted on Twitter, and we used Twitter posts, Twitter messages, and tweets interchangeably. We then removed all retweets, defined as messages that had been shared and circulated by users other than the initial poster. Finally, we used an inductive qualitative coding approach to code Twitter messages from manually selected clusters that contained word groupings related to the study aims.

### Data Collection and Processing

We collected data from Twitter using the public streaming API over a period of approximately 3 months from March 3, 2020, to Oct 28, 2020, using the Python package Tweepy (version 3.8.0) [16]. This period was selected because it marked the acceleration of the outbreak into a global pandemic and was a crucial period for the establishment of pro- and anti-vaccination sentiment, as vaccine development was widely discussed and debated. The data included the text of the Twitter messages and other metadata associated with the message (eg, geolocation, if available; time stamp information; and user account or handle). We first applied a list of common COVID-19–related keywords used on social media as filters for the Twitter public API. These keywords were chosen on the basis of structured manual searches conducted on Twitter that detected content related to the COVID-19 pandemic as posted by users, and they had also been validated as being able to identify tweets pertaining to general COVID-19 conversations in prior studies [17-21]. These keywords included “coronaoutbreak,” ”corona,” ”anticorona,” “coronavirus,” “covid,” and “pandemic.” We captured and processed all Twitter messages that contained at least one of these keywords or hashtags. The purpose for this first phase of keyword selection was to obtain a broad Twitter corpus that contained general COVID-19–related conversations not specific to any topic, which could then be filtered for more specific hashtags, keywords, and other vocabulary associated with anti-vaccination sentiment, opinions, or statements.

After removing duplicate tweets, we applied a second text filter to isolate tweets with anti-vax–related keywords and conducted BTM. Anti-vax–related keywords included “antivaxxing,” “antivaxx,” “antivaxxers,” “antivax,” “anti-vaxxer,” “discredit,” “undermine,” “confidence,” and “immune.” We chose anti-vax–related keywords, as we were specifically interested in the web-based discourse surrounding these terms, and these terms also appeared as related search terms when conducting testing of related terms associated with “anti-vaccine” on the Google search engine. For the purposes of our analysis, “anti-vax” is equivalent to anti-vaccination, and therefore, the 2 terms are used interchangeably. Our investigation defines vaccine deniers, more commonly referred to as “anti-vaxxers,” as individuals who believe vaccines are dangerous, deny the efficacy of inoculation, or refuse vaccines for themselves and their children, if applicable.

### Topic Modeling Using BTM

BTM groups Twitter messages containing the same word-related themes and summarizes the entire corpus of text into distinct highly correlated categories. BTM is best used for short text, and its primary strengths are topic modeling word co-occurrence patterns and identifying such sequences in text that contain few words [15]. The main themes in clusters produced by BTM are considered an aggregation of topics from the text, which are then split into a bag of words, where a discrete probability distribution for all words in each theme is generated. Before running BTM, we cleaned our data set for imbedded hyperlinks, stop words, special characters and punctuation marks, and length using the Natural Language Toolkit package in Python [22]. Specifically, we excluded Twitter posts less than 3 words in length, as they likely do not convey sufficient information for purposes of inductive content coding of themes, which is consistent with prior studies [23]. Using the COVID-19 data set filtered for the anti-vax–related keywords described, we used BTM to parse the data into 20 topic clusters.

We set a total number of 20 different clusters (ie, total number of topics for BTM to output: k=20), resulting in texts with similar themes put into the same clusters. To find the appropriate k value, we used a topic coherence score [21,24]. Coherence score is used to measure the performance of a topic model with different number of clusters and can help differentiate between topics that are semantically interpretable and topics that are artifacts of statistical inference [24,25]. We tested 5 different k values (k=10, 20, 30, 40, and 50) for each data set and found that when k=20, we generated the highest coherence score, and this score did not change significantly with an increase in the k value.

### Screening

We manually screened the top 10 tweets that were most highly correlated to the 20 topic cluster word groupings generated following the BTM topic modeling phase. By examining the top 10 tweets, we ensured that we did not miss public figures mentioned in other topics. In BTM, correlation is determined by word co-occurrence patterns in the text, and the outputted clusters were then manually reviewed for relevance. We then manually selected 5 clusters that most closely included messages calling out or making claims about public figures as anti-vaxxers or that called out groups of people such as scientists or political parties. We define a public figure as a Twitter user with a verified Twitter account. Topic clusters that were not included in this study covered topics about government mistrust, conspiracies, mask business promotion, and general statements about anti-vaccination beliefs (not specific to any public figure).

### Content Analysis

Our sample included Twitter messages associated with these 5 relevant clusters outputted by BTM and then extracted for all tweets associated with the selected clusters. We applied a grounded theory’s inductive coding approach, allowing for themes to emerge while coding rather than prespecifying the content of interest [26]. Grounded theory enables researchers to develop a theory to explain the phenomenon of interest, and as the study progresses, the researcher’s initial exploratory question becomes refined until an understanding is reached regarding the topic of investigation [27]. The advantage of this approach is that it allows us to minimize the effect of personal bias surrounding vaccination rhetoric [26] by generating the codes based on the content of the Twitter messages. We conducted qualitative analysis to characterize the discourse (eg, pro-vaccination vs anti-vaccination). After the first round of manual review, we inductively developed a codebook for the qualitative content analysis and categorization of Twitter posts. We then reapplied our codebook to the Twitter messages in our sample, while iteratively continuing to develop existing codes and definitions as well as new codes.

We also labeled the public figure or group mentioned in each post, where applicable, and calculated the corresponding frequencies and percentages (Table 1). We reached thematic saturation after approximately 200 posts but continued to code the entire data set. Three of the authors (MS, US, and NR) coded the Twitter messages independently and achieved a high intercoder reliability (κ=0.92). For inconsistent results, all coders met and conferred on correct classification and subclassifications to reach consensus. Coders denoted neutral, anti-vaccination, or pro-vaccination sentiments expressed in the messages, along with each theme, throughout 7 rounds of coding.

**Table 1:** Public figures or groups mentioned in selected sample of Twitter messages sorted by frequency (n=768).

Public Figure^a^	Frequency^b^, n (%)^c^
Novak Djokovic	330 (43)
None	149 (19.4)
Kamala Harris	148 (19.3)
Joe Biden	123 (16)
Donald Trump	20 (2.6)
Amy Duncan^d^	13 (1.7)
Isabel Lucas	12 (1.6)
Andrew Cuomo	9 (1.2)
Barack Obama	7 (0.9)
Ammon Bundy	6 (0.8)
Joe Rogan	5 (0.7)
Bill Gates	5 (0.7)
Rand Paul	5 (0.7)
Rebecca Judd	5 (0.7)
Alex Jones	4 (0.5)
Anti-vaxxers^d^	3 (0.4)
Kanye West	3 (0.4)
Jim Carrey	3 (0.4)

^a^We analyzed Twitter messages collected using anti-vaccination hashtags involving public figures or groups. The public figures or groups mentioned in these messages do not all explicitly express anti-vaccination ideology but have been included in analysis for the assessment of Twitter rhetoric surrounding these individuals or entities.

^b^Public figures or groups mentioned ≤2 times were excluded from the table. Excluded public figures or groups are as follows: Washington Post, Jeanine Piro, University of Toronto Scarborough, Carolyn Maloney, Tommy Robinson, Scientists, David Icke, Russian government, Robert Redfield, Qanon, Ian Brown, White House Coronavirus Taskforce, Dejan Lovren, Catholic Archbishops, Jim Acosta, Sebastian Gorka, Mike Lindell, Sharyl Attkisson, Leigh-Allyn Baker, Nikola Jokic, Rita Pala, Lee Zeldin, Bernie Sanders, Judy Mikovits, John Water, Federal Agencies, Democrats, online anti-vax communities, MIA, British Union of Fascists, Glenn Davies, George Stephanopoulos, and Marianne Williamson.

^c^Authors assigned multiple public figures or groups to various Twitter messages, when applicable; therefore, percentages do not add up to 100%.

^d^Amy Duncan is a fictional character, and anti-vaxxers are a group. We manually selected Biterm Topic Modeling clusters based on relevance to public figures, which contained figures and groups with both verified and unverified accounts.

## Results

### Overview

We collected the initial sample from various anti-vax–related keywords, and the sample contained 3,999,726 Twitter posts. We then removed all duplicate tweets with the same tweet ID that distilled our sample to 118,971 messages. Subsequently, we grouped Twitter posts into topic clusters using BTM, yielding 20 clusters containing various topics. We then selected 5 clusters most closely related to public figures, anti-vaccination, and anti-lockdown. We manually reviewed a cumulative sum of 768 Twitter messages identified in the 5 topic clusters selected.

Of the 768 Twitter messages, 425 (55%) were neutral, 329 (43%) expressed pro-vaccination sentiments, and 14 (2%) expressed anti-vaccination sentiments. Furthermore, 356 (46%) messages called out public figures for their stances or behaviors, 188 (24%) undermined public figures, 157 (20%) expressed concern over the negative public health impact of the actions of certain public figures, 57 (7%) insulted public figures, and 8 (1%) defended anti-vaccination public figures (Table 2). A total of 51 public figures with verified Twitter accounts were identified comprising a mix of athletes, politicians, actors, musicians, radio and political commentators, models, business leaders, anti-government activists, and other personalities. Politicians were some of the most frequently mentioned public figures, along with political commentators.

**Table 2:** Twitter message themes.

Theme	Definition	Example	Frequency, n (%)
Neutral or none	Absence of expression of a clear judgement even if the message is related to the topic [18].	*Latest on our #globalhealth #COVID19 #vaccine. Note to my antivax friends: this is not Merck & Co the vaccine company, it’s Merck Darmstadt in Germany, which owns Sigma Millipore, totally separate*	2 (0.3)
Insults	Insults a person because they are an anti-vaxxer; says something derogatory to someone because they are or have been accused of being an anti-vaxxer.	*Wouldn’t it be tragic if anti-vaxxer idiot Novak Djokovic succombs* [sic] *to coronavirus before a vaccine he refuses to take is invented? *	57 (7.4)
Negative public health impact	States or implies that anti-vaxxers and anti-vaccine behaviors have a negative impact on public health. May connect the vaccine to other diseases.	*Opinion | Anti-lockdown and anti-vaxxer protesters have merged. And it could be deadly. via @....*	157 (20.4)
Anti-vax accusations	Accuses or asserts a specific person or groups of people are anti-vaxxers. Subcode of “undermine”: accusations intending to undermine or discredit a person or group(s).	*Trump calls Dems anti-vaxxers says their anti-vaccine rhetoric is ‘dangerous’ after Sen. Harris said she wouldn't trust the him* [sic] *on safety of COVID vaccine before the election.*	188 (24.5)
	Accuses or asserts a specific person or groups of people are anti-vaxxers. Subcode of “call-out”: accuses a person or group, call them out, or imply that they are against vaccines.	*Tennis: Novak Djokovic is an anti-vaxxer and won’t take coronavirus vaccine #tennis #tennisnews*	356 (46.4)
Defending anti-vax stance	Defends or upholds an anti-vax position.	*Novak Djokovic's wife is shamed with a 'False Information' badge by Instagram for spreading coronavirus 5G conspiracy theories after the tennis world No 1 revealed he's an anti-vaxxer….Stand strong Jelena!*	8 (1)

### Theme 1: Anti-Vax Accusations

Twitter users frequently accused public figures of holding anti-vaccination views. Twitter posts that “called out” or accused public figures of harboring anti-vaccination beliefs composed the largest segment of our sample (356/768, 46%). In most cases, these posts refered to public figures who publicly espoused anti-vaccination attitudes, such as Novak Djokovic. Messages intending to undermine or discredit public figures formed 24% (188/768) of our sample, with the majority expressing neutral sentiment toward vaccination. Some of these messages amplified statements made by public figures that undermined or accused other public figures of harboring anti-vax beliefs, which was common among politicians in our sample. Specifically, these messages amplified statements made by the former US president Donald Trump accusing Joe Biden and Kamala Harris of subscribing to anti-vax beliefs. Notably, the majority of undermining messages in our sample mentioned Kamala Harris and Joe Biden.

### Theme 2: Insults

Our sample included 57 (7%) Twitter messages insulting public figures. The vast majority of insults were directed toward public figures suspected of being anti-vaxxers or toward public figures providing known anti-vaxxers with a platform to voice their ideologies. Similar to undermining, these messages attempted to discredit anti-vaxxers by degrading their beliefs using derogatory terms; however, these insults differed from undermining messages, as the latter accused or implied an anti-vaccination stance, whereas the former blatantly disrespected public figures with demeaning remarks. Of the 57 messages insulting public figures, 35 (61%) were directed at Novak Djokovic. Overall, Twitter messages containing insults targeted a broad scope of public figures, with the majority either known or suspected to be holding anti-vaccination beliefs.

### Theme 3: Negative Public Health Impact

Our sample contained 157 (20%) Twitter messages stating or implying that anti-vaccination behaviors or rhetoric expressed by public figures may have a negative public health impact. These messages typically expressed concern about the effects of the anti-vaccination movement on public health. Of these messages, 88 (56%) expressed a neutral attitude toward vaccination, while 69 (44%) explicitly expressed pro-vaccination sentiments. The majority of Twitter messages (116/157, 74%) characterized as expressing a belief that anti-vaccination rhetoric or behaviors stemming from public figures have a negative public health impact were not directed toward specific public figures, but rather targeted anti-vaxxers in general.

## Discussion

Given the prevalence of anti-vaccination attitudes and the known contagion of celebrity beliefs, we expected to see mentions of public figures with anti-vaccination beliefs further espousing vaccine misinformation sentiment and conspiracies on the internet; instead, we found that “anti-vax”–related keywords or hashtags in our corpus of tweets primarily consisted of discourse accusing or insulting public figures of holding an anti-vaccination stance, specifically as a means of publicly calling them out or insulting them on Twitter. Notably, the majority of Twitter messages (56/57, 98%) characterized as insults expressed pro-vaccination sentiment, indicating that insults are frequently sent by supporters of vaccination rather than anti-vaxxers.

There were multiple posts accusing public figures of holding anti-vaccination beliefs (including known vaccine supporters). Undermining messages attempt to discredit public figures by accusing them of holding anti-vax beliefs, even among those known to publicly support vaccines. These posts exemplify the denunciation of suspected anti-vaxxers by Twitter users [28,29].

As expected, an abundance of posts insulted public figures known to be anti-vaxxers, and a recent systematic review examining misinformation found it to be a universal source of stress, fatigue, insomnia, and anger [30]. Further research should focus on identifying common traits among public figures subject to insults from social media users and the effect of this overall rhetoric on other users’ attitudes, knowledge, and perceptions about vaccines.

Our study has several limitations. First, we acknowledge that attitudes of Twitter users are unlikely to be representative of the general population or attitudes specifically toward celebrities or public personalities. Second, we sampled based on anti-vaccination keywords and for a specific period of time during the COVID-19 pandemic; this method is a common approach in infodemiology and misinformation studies [13,31,32], but we could have nevertheless missed messages relevant to the study aims that did not include these hashtags or occurred later during the pandemic. Hence, our choice of keywords or hashtags used for this study is not generalizable to all anti-vax posts occurring on Twitter. Third, we performed content analysis with a circumscribed sample of tweets outputted by topic modeling; there may be additional themes linking public figures and vaccination that did not emerge in our sample.

For the majority of our sample, referring to a public figure as an “anti-vaxxer” is a way of condemning public figures, whether or not they espouse anti-vaccination beliefs in their own public communication. Novak Djokovic openly opposes vaccination, but pro-vaccination individuals, including President Biden, have been accused of being “anti-vax” on Twitter. This unexpected finding in the context of user-generated posts associated with anti-vax–related keywords and hashtags (ie, we expected to observe amplification of anti-vax sentiment harbored by known celebrities and public figures) suggests reciprocal influence between public health recommendations and attitudes about public figures rather than the previously described one-way, outsize influence of celebrities on vaccination attitudes. We believe that social media platforms represent a complex information ecosystem, where anti-vax sentiment may not reside in common anti-vax–related keywords or hashtags, but instead in other web-based spaces of discourse that require additional study. Additional research is also needed to fully assess the influence of public figures and users’ perception of these individuals on ensuing vaccine discourse, whether positive or negative.

## Abbreviations

BTM: Biterm Topic Modeling
API: application programming interface

## Data Availability

Deidentified data that contains associated twitter IDs associated with this study are available in a GitHub repository [33].
